# An ELISA-like sensitive and visual detection system targeting *Yersinia pestis* based on CRISPR/Cas12a and DNAzyme

**DOI:** 10.1128/jcm.00274-25

**Published:** 2025-07-24

**Authors:** Yingqing Mao, Ruichen Lv, Hao Shao, Yong Zhao, Junhu Wang, Qiong Chen, Haiming Yi, Yixin Ge, Hongming Wang, Yuexi Li, Yong Qi

**Affiliations:** 1 Huadong Research Institute for Medicine and Biotechniques, Nanjing, China; 2 Teachers College, Columbia University 5930, Manhattan, New York, USA; 3 State Key Laboratory of Pathogen and Biosecurity, Beijing Institute of Microbiology and Epidemiology, Beijing, China; Endeavor Health, Evanston, Illinois, USA

**Keywords:** *Yersinia pestis*, CRISPR-Cas12a, recombinase-aided amplification, G-quadruplex, nucleic acid detection, diagnosis

## Abstract

*Yersinia pestis* is the causative agent of plague, a human disease with potentially devastating consequences. Here, we developed an enzyme-linked immunosorbent assay-like visual detection method based on clustered regularly interspaced short palindromic repeats (CRISPR) detection and DNAzyme for the cost-effective and highly sensitive detection of *Y. pestis*. A novel specific gene sequence (CH57_3927) was screened for the detection target of *Y. pestis*. The recombinase-aided amplification (RAA) assay, CRISPR/Cas12a detection assay, and G-quadruplex (G4) DNAzyme-based color development assay were separately established and optimized. These three optimized assays were integrated into an advanced ELISA-like visual detection method—RAA-CRISPR/Cas12a-DNAzyme (RCCD)—by further optimization of their components to improve the compatibility between them. The amplified target sequence binds to crRNA and activates the Cas12a nucleases for trans-cleave G4. As a result, the cleaved G4 is unable to bind with hemin to exert peroxidase activity, thus impeding the catalysis of the 2,2'-azino-bis(3-ethylbenzothiazoline-6-sulfonic acid) (ABTS^2–^) colorimetric reaction. Consequently, negative samples exhibit a dark green coloration, while the positive products appear nearly colorless, facilitating visual differentiation with the naked eye. In addition, the RCCD detection platform effectively distinguished *Y. pestis* from all other closely related species, with a detection limit of 1 copy/reaction. Evaluated using *Y. pestis* DNA-spiked blood samples and uninfected samples, both sensitivity and specificity were 100%. The method shows significant potential for detecting targets in clinical samples and is well-suited for use in resource-limited environments. It offers advantages such as visual detection, batch detection, and low cost.

**IMPORTANCE:**

We utilized Mauve software to screen *Yersinia pestis* specific genes and integrated CRISPR-Cas12a, RAA amplification, and G-quadruplex DNAzyme technology to establish an advanced ELISA-like visual detection method. The visual detection method offers a more cost-effective alternative compared to the conventional CRISPR detection method that relies on fluorescence-labeled ssDNA reporter or lateral flow (LF) test strips. With only one thermostatic device required, it enhances the convenience of rapid on-site screening of *Y. pestis* outbreaks, providing effective support for plague detection, prevention, and control within primary medical and health institutions.

## INTRODUCTION

Plague is a deadly infectious disease caused by *Yersinia pestis*, known for its rapid transmission and high fatality rate (1). Throughout history, three pandemics of plague have resulted in over 100 million deaths. According to statistics from the World Health Organization’s official website and reports from various countries, a total of 58,752 cases of plague were reported globally between 1987 and 2020, including 4,888 deaths (2–4). Since the 21st century, more than 95% of plague cases have been concentrated in Africa, with Congo, Madagascar, and Peru emerging as the three most heavily impacted countries (5, 6). In August 2017, a severe pulmonary plague epidemic broke out in Madagascar. During the period from 1 August to 26 November 2017, 2,417 cases were recorded, including confirmed, possible, and suspected cases, along with 209 deaths (7). The prevalence of plague remains a challenge to public health. Therefore, it is crucial to develop a rapid and sensitive diagnostic method for plague.

Traditional methods for detecting *Y. pestis* involve isolating and culturing the bacteria from clinical samples, conducting phage lysis assays, and utilizing serological detection methods that rely on identifying antibody-mediated F1 antigens, such as enzyme-linked immunosorbent assay (ELISA) (8), passive hemagglutination assay (PHA) (9), and fluorescent antibody (FA) assay (10). However, these conventional bacteriological detection techniques are prone to misjudgment with other bacteria in the *Enterobacteriaceae* family (11). Furthermore, they are time-consuming and require costly equipment. Consequently, there is an imperative need to develop a rapid, uncomplicated, and efficient diagnostic method for *Y. pestis* detection in resource-limited environments.

Nucleic acid detection methods offer a promising alternative for detecting and distinguishing *Y. pestis*, allowing for the differentiation of *Yersinia* species closely related to *Y. pestis*. Nucleic acid detection methods such as real-time fluorescent PCR (RT-PCR), recombinase polymerase amplification (RPA), recombinase-aided amplification (RAA), and loop-mediated isothermal amplification (LAMP) have played crucial roles in the diagnosis of infectious diseases. However, the practical application of pathogen detection puts forward a higher demand for detection simplicity and detection sensitivity. The clustered regularly interspaced short palindromic repeats (CRISPR) detection technology is a newly developed technique in recent years. During the COVID-19 pandemic, CRISPR detection technology underwent rapid development, with multiple COVID-19 virus detection reagents based on this technology being approved (12). The basic principle relies on the trans-cleavage characteristics of the Cas proteins in the CRISPR system. It involves designing crRNA that is complementary to the target nucleic acid sequence to be detected. The Cas protein binds with the crRNA to form a complex, which recognizes and binds to the target nucleic acid sequence through complementary pairing, thereby activating the trans-cleavage activity of the Cas protein in the system (13). This, in turn, cleaves the reporter molecules in the system that carry fluorescent reporters and quenchers, releasing detection signals for molecular diagnosis (14). The combination of RAA and CRISPR/Cas12a has been successfully used for the detection of various pathogens, such as hepatitis B virus (HBV) (15), severe acute respiratory syndrome coronavirus 2 variables (SARS-CoV-2 variables) (16, 17), African swine fever virus (ASFV) (18), *Vibrio vulnificus* (19), *Vibrio parahaemolyticus* (20), and *Listeria monocytogenes* (21).

DNAzyme is a class of DNA molecules with catalytic functions. Activated Cas12a can trans-cleave single-stranded DNA and inactivate the catalytic functions of DNAzyme. Therefore, it is suitable for combining Cas12a and DNAzyme to build a biosensor. A commonly used DNAzyme, G-quadruplex (G4), is an intricate structure formed by stacking single-stranded DNA rich in guanine and stabilized by monovalent cations like K^+^ and Na^+^ (22, 23). The G4 DNAzyme demonstrates exceptional thermodynamic resilience, maintaining structural integrity and catalytic activity across a wide thermal gradient, which ensures reliable chromogenic performance under diverse environmental conditions. Furthermore, the colorimetric reaction between G4 and hemin is more cost-effective than traditional signal reporting systems, such as fluorescence and lateral flow (LF) test strips, while generating a robust colorimetric signal that is easily visible to the naked eye. G4 is known for its simple synthesis, cost-effectiveness, and robust thermal stability in the design and development of highly sensitive molecular diagnostic methodologies. It has been used for signal transduction and target recognition and further developed into G4-based biosensors with easy operation and visualization capabilities (24–26). The interaction of G4 with specific fluorescent ligands can significantly amplify its fluorescence signal and be used as a fluorescent tracer in constructing highly sensitive fluorescent biosensors (27–30). The formation of DNAzymes by combining G4 with hemin has the characteristics of inducing color changes in substrates, catalyzing luminol-H_2_O_2_ chemiluminescence reactions, and quenching the fluorescence signals of nanoparticles. Based on these characteristics, G4 DNAzyme has been used to develop biosensing platforms for various detection methods, such as electrochemistry (31), chemiluminescence (32), fluorescence detection (33), and colorimetry (34). Therefore, it is supposed that combining Cas12a with G4 in a liquid detection system will greatly reduce the detection cost and improve the detection throughput of the CRISPR-based visual detection.

The aim of this study was to develop a sensitive, highly specific, rapid, and cost-effective *Y. pestis* detection method based on CRISPR/Cas12a and G4 DNAzyme technologies. Our results suggest that the RAA-CRISPR/Cas12a-DNAzyme (RCCD) detection platform holds promise for successful implementation in clinical environments.

## MATERIALS AND METHODS

### Materials and reagents

RAA nucleic acid amplification kit (basic version) and RAA nucleic acid amplification kit (fluorescent method) were purchased from Jiangsu Qitian Gene Biotechnology Co., Ltd. (Wuxi, China). LbCas12a nuclease was purchased from GenScript (Nanjing, China). NEBuffer r2.1 was purchased from New England Biolabs (Beijing, China). Hemin was purchased from Solarbio (Beijing, China). EL-ABTS Chromogenic Reagent kit (containing 2,2'-azino-bis(3-ethylbenzothiazoline-6-sulfonic acid), ABTS) and 3,3',5,5'-tetramethylbenzidine (TMB) Chromogen Solution (for ELISA) were purchased from Sangon (Shanghai, China). Dithiothreitol (DTT) was purchased from Beyotime (Shanghai, China). H_2_O_2_ was purchased from HANNO (Huainan, China). Plasmids, crRNAs, primers, reporters, and G4 ssDNA were chemically synthesized by Sangon company, and the DNA and RNA sequences used in the work are listed in Table 1. All other chemical reagents were purchased from Sangon company. Genomic DNAs of *Y. pestis* (*Microtus* 201) and other bacterial species (Table 2) were extracted from the corresponding strains using a QIAwave DNA Blood & Tissue Kit (QIAGEN, Hilden, Germany). The concentrations of the extracted DNAs were determined according to the absorbance at a wavelength of 260 nm measured by the microspectrophotometer (KAIAO, K5600). All genomic DNAs were diluted with elution buffer to 10 ng/µL. Fifteen blood samples were collected from blood donors in a hospital for this study. The use of these samples was reviewed and approved by the Ethics Committee of the Huadong Research Institute for Medicine and Biotechniques (approval no. 2022005).

**TABLE 1:** Oligonucleotide sequences employed in this work^*a*^

Names	Sequence (5′−3′)
CH57_3927-F2	GCATAAGCTCTTGTAGTAACTCTGGCGATAT
CH57_3927-F3	AACTCTGGCGATATTTTGTCGGGGTAGTAAT
CH57_3927-F6	CTCTTGTAGTAACTCTGGCGATATTTTGTCGG
CH57_3927-R2	CTCAACATGACGAGCAATTAGTGGTATGTGGC
CH57_3927-R3	TAGTGGTATGTGGCGTTCCTATCGGTGAA
CH57_3927-R4	TATTGAAACCTGCTTTCCTTATGCCTCTG
CH57_3927-R5	ATGTGGCGTTCCTATCGGTGAAGGGAGTAT
CH57_3927-R6	TCCTTATGCCTCTGCATTTTCTCGACCTTG
CH57_3927-R7	TGAAAAATCAGGCATAACTCGGGTCAATAT
CH57_3927-R8	GATCACATTGTTACTCAACATGACGAGCAA
CH57_3927-R9	TTACTTACAGTACCCATTTTAAAGATCACA
CH57_3927-P1	TAATTCGGCTGGAGTCAAAAGGAGGGGTT/i6FAMdT//idSp//iBHQ1dT/ACTTCAAAAGTCCC
CH57_3927-crRNA1	UAAUUUCUACUAAGUGUAGAUUCGGGGUAGUAAUAUUCCAGUGGUU
CH57_3927-crRNA2	UAAUUUCUACUAAGUGUAGAUAUUCGGCUGGAGUCAAAAGGAGG
CH57_3927-crRNA3	UAAUUUCUACUAAGUGUAGAUCUUCAAAAGUCCCUUUUAGUGUA
CH57_3927-crRNA4	UAAUUUCUACUAAGUGUAGAUGUGUACUGACUUCAUUGGGGUUG
CH57_3927-crRNA5	UAAUUUCUACUAAGUGUAGAUAAUAUUGACCCGAGUUAUGCCU
CH57_3927-crRNA6	UAAUUUCUACUAAGUGUAGAUAACCAAUAUUCUGAAGCAAUUA
CH57_3927-crRNA7	UAAUUUCUACUAAGUGUAGAUAAAUGGGUACUGUAAGUAAAGGG
CH57_3927-crRNA8	UAAUUUCUACUAAGUGUAGAUCUAUAUCGCAAUGUGCCCCGAC
CH57_3927-crRNA9	UAAUUUCUACUAAGUGUAGAUGAUAAUCUCGUCGGCAGUUUC
CH57_3927-crRNA10	UAAUUUCUACUAAGUGUAGAUCCUGAAGUAGAGGAACCAGUGAU
CH57_3927-crRNA11	UAAUUUCUACUAAGUGUAGAUAACUCAUGAAUAAUGCGUGGAU
CH57_3927-crRNA12	UAAUUUCUACUAAGUGUAGAUCUAAUCGCUUUAACCGACGUA
FAM-12T-BHQ1 reporter	5′6-FAM -TTTTTTTTTTTT-BHQ1-3′
G-rich ssDNA-1	CTGGGAGGGAGGGAGGGA
G-rich ssDNA-2	TTAGGGTTAGGGTTAGGGTTAGGGTTA
G-rich ssDNA-3	TTTGGGAAGGGCGGGTAGGGT
G-rich ssDNA-4	TGGGTAGGGCGGGTTGGGAAA
G-rich ssDNA-5	GGTTGGTGTGG
FAM-G-rich ssDNA-4-BHQ1	5′6-FAM -TGGGTAGGGCGGGTTGGGAAA-BHQ1-3′

^*a*^ The complementary sequences are indicated by underline.

**TABLE 2:** Bacterial species used in this study

Species	Strains	Sources
*Yersinia rohdei*	43380	ATCC
*Yersinia mollaretii*	43969	ATCC
*Yersinia kristensenii*	33638	ATCC
*Yersinia frederiksenii*	33641	ATCC
*Yersinia ruckeri*	29473	ATCC
*Yersinia intermedia*	29909	ATCC
*Yersinia pseudotuberculosis*	28933	ATCC
*Yersinia enterocolitica*	9610	ATCC
*Yersinia pestis*	*Microtus* 201	Our laboratory
*Escherichia coli*	O157:H7	Our laboratory
*Staphylococcus aureus*	25923	ATCC
*Pseudomonas aeruginosa*	10145	ATCC
*Bacillus subtilis*	6051	ATCC
*Bacillus thuringiensis*	10792	ATCC
*Vibrio parahaemolyticus*	17802	ATCC

### Specific gene sequences screening

To screen specific DNA fragments of *Y. pestis*, the genome sequences of all species within the genus *Yersinia* were collected and aligned in Mauve version 20150226 (The Darling lab at the University of Technology Sydney) (35). All the specific sequences of over 300 bp in *Y. pestis* were selected and further aligned with all the public genome sequences of *Y. pestis* strains using BLAST software online (http://blast.ncbi.nlm.nih.gov/) to ensure the sequence is conserved within the species. To test the out-of-genus specificity of the selected sequences, they were aligned with all the gene sequences from non-*Yersinia* species in GenBank. The sequence showing the highest specificity and conservation was selected as the target gene, chemically synthesized by Sangon Biotech Company, and linked to pUC57 plasmid to construct a positive template for further detection method development.

### Cas12a-based detection method development

A series of crRNA sequences were designed according to the restricted PAM sequences for Cas12a and chemically synthesized by Sangon. Each crRNA featured a 21 bp universal sequence (5′-UAAUUUCUACUAAGUGUAGAU-3′) (16) and a complementary sequence to the target gene. The initial Cas12a-based detection system was formulated with 20 nM of LbCas12a, 100 nM of crRNA, 50 nM of FAM-TTTTTTTTTTTT-BHQ1 reporter, and the positive plasmids (10^10^ copies). All the components were mixed in 20 µL of 1 × NEB buffer 2.1 and incubated at 37°C. Real-time fluorescence signals were measured by a F1620 fluorescent reader (Qitian Gene Biotechnology Co., Ltd.) at 20-s intervals for an hour. A negative control using plasmid pUC57 as the template was conducted in each test. The dynamic change of fluorescence value with reaction time was plotted, and the slope was calculated. A higher slope indicated a higher detection efficiency. The optimal crRNA was selected with the highest slope ratio between the positive group and the negative group.

### Optimization of Cas12a-G4 colorimetric reaction development

Five experimental conditions were optimized, including the concentrations of G4, Cas12a, and hemin, the types of color-developing substrates, and the sequence of G-rich ssDNA. First, the concentration ratio of Cas12a to G4 was optimized by testing various Cas12a concentrations (223 nM and 111 nM) and G4 concentrations (0.25 nM, 0.5 nM, and 1 nM). The colorimetric reaction involved testing different concentrations of Cas12a and G4, 100 nM crRNA, and the positive plasmids (10^10^ copies). All the components were mixed in 20 µL of 1× NEB buffer 2.1 and incubated at 37°C for 2 hours. After incubation, 1 µL of hemin (50 µM) and 2 µL of KCl (500 mM) were added to activate the peroxidase activity. Then, 50 µL of the EL-ABTS chromogenic reagent was added, and the mixture was incubated at room temperature for another 10 min. The color change of the solution was observed, and the absorbance was measured by a Spark microplate reader (TECAN, Männedorf, Switzerland). The optimal concentration of Cas12a and G4 was determined based on visual color changes and absorbance differences between the positive plasmid and plasmid-free control groups.

Subsequently, the hemin concentration and the type of chromogenic agent were optimized following the above experimental procedure. Specifically, experiments were conducted with varying hemin concentrations (2.5 µM, 5 µM, and 10 µM) and different chromogenic reagents (ABTS and TMB). The optimal experimental conditions were determined based on visual color observation and absorbance differences. Finally, under the optimal conditions established above, G-rich ssDNAs with various DNA sequences were tested to identify the G4 sequence with the best color rendering effect.

### RAA assay development

To develop an RAA assay that can be combined with the Cas12a-G4 reaction, a series of RAA primers were designed on either side of the optimal crRNA site in the target sequence according to the manufacturer’s instruction. An RAA probe was also designed with FAM and BHQ1 labeled. An RAA nucleic acid amplification kit (fluorescent method) was used to evaluate the gene amplification efficiency of each primer pair. In the RAA reaction system, 4.2 µL of each primer pair (10 µM), 0.6 µL of RAA probe (10 µM), 1 µL of template, and 25 µL of buffer were mixed and complemented with ddH_2_O to 47.5 µL. The reaction mixture was then transferred into the tube containing the lyophilized RAA enzyme mix. Subsequently, 2.5 µL of magnesium acetate was added to initiate the reaction, and the tubes were placed into the B6100 Oscillation mixer (Qitian Gene Biotechnology Co., Ltd.) for pre-amplification for 4 min. The tubes were moved to the F1620 fluorescent reader for fluorescence measurements at 20-s intervals over 20 min at 37°C. The optimal primer pair was selected with the highest reaction efficiency.

### RCCD detection assay development and preliminary evaluation

The optimized RAA assay was combined with the Cas12a-G4 reaction to construct a visual detection method. Briefly, the target fragment was amplified using the optimized RAA assay. Here, the RAA nucleic acid amplification kit (basic version) was used without the probe added. Then, various volumes of the amplification product were added to the optimized Cas12a-G4 colorimetric reaction system for colorimetric detection. Genomic DNAs of various *Yersinia* species were used as templates in the constructed RCCD detection assay for specificity evaluation. In addition, to evaluate the limit of detection (LOD) of the assay, serially diluted genomic DNA (with concentrations of 10^3^, 10^2^, 10, and 1 copies/µL) of *Y. pestis* in elution buffer was used as templates, and the minimum concentration that could be detected was the LOD. Elution buffer served as the template of negative control during both experiments. In the experiments above, each reaction was performed with at least two replicates.

### RCCD detection assay evaluation using simulated clinical samples

The detection performance of the established assay was evaluated using simulated clinical samples due to the unavailability of clinical samples from *Y. pestis*-infected patients. *Y. pestis* genomic DNA was added to 200 µL of uninfected blood samples, achieving final concentrations of 30 copies/µL. DNA was subsequently extracted from the simulated clinical samples using the QIAamp DNA Blood & Tissue Kit, with a final elution volume of 200 µL. The RCCD detection assay was then performed on the extracted DNA. The original blood samples served as the negative controls, with each test conducted in duplicate.

### Data processing and statistical analysis

The UV-visible absorption curve was plotted using Origin 2018 software (OriginLab, Northampton, MA, USA). The OD values among various groups were compared using one-way ANOVA or two-way ANOVA using GraphPad Prism 8.3.0 software (GraphPad Software, Boston, MA, USA), and the difference was considered significant with a *P* value of <0.05.

## RESULTS

### Detection principle

**Fig 1:**
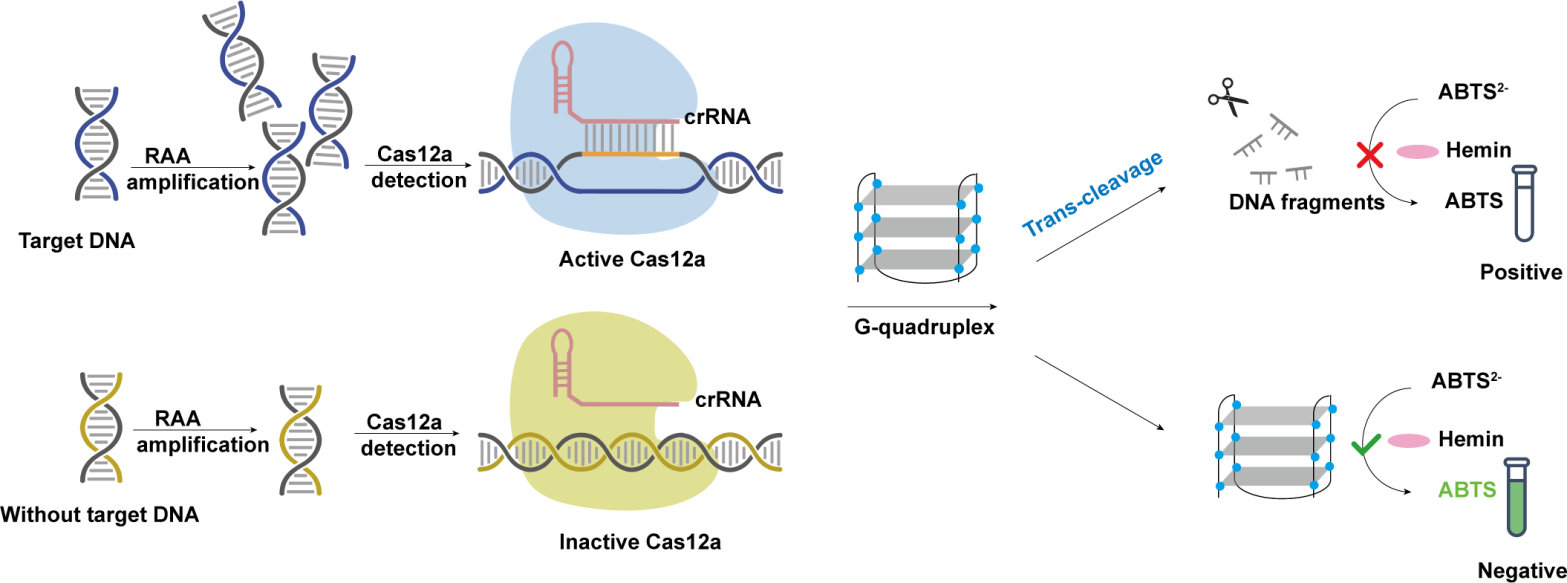
Schematic illustration of the visual detection system of *Y. pestis* combining RAA, CRISPR/Cas12a, and G-quadruplex DNAzyme.

The principle of the RCCD detection platform is shown in Fig. 1. The target gene fragment is initially amplified through RAA amplification. The amplified target sequence binds to crRNA and activates the Cas12a nucleases for trans-cleave G4. Consequently, the cleaved G4 is unable to bind with hemin to exert peroxidase activity, thus impeding the catalysis of the ABTS^2–^ colorimetric reaction. The color of the solution will remain transparent. Conversely, in the absence of the target gene, G4 will not be cleaved and can bind with hemin to form G4/hemin DNA enzyme, catalyzing the color reaction of ABTS^2–^ and resulting in a color change from transparent to blue-green. The colorimetric signal could not only be detected by an ELISA reader but also observed by the naked eye.

### Specific gene sequence screening

A novel specific gene named CH57_3927 (nucleotides from 4338643 to 4339629 bp in *Y. pestis* A1122 references CP009840.1) of *Y. pestis* was successfully identified as the target. As shown in Fig. S1, this sequence was shared by all 81 strains of *Y. pestis* but not by any other *Yersinia* species or public sequences, indicating that it is an ideal target sequence for developing nucleic acid detection methods.

### Optimal crRNA screening for Cas12a detection

**Fig 2:**
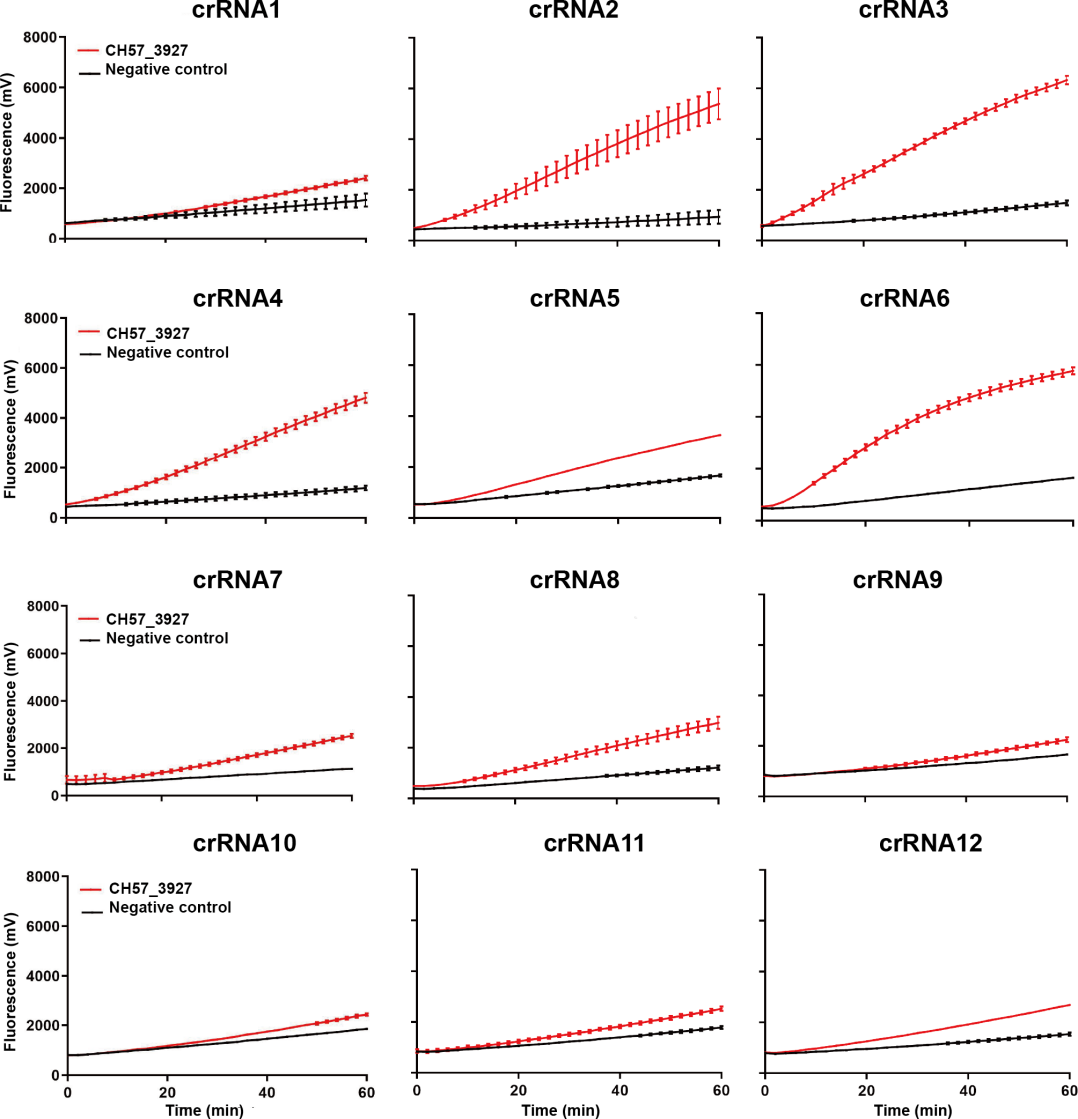
crRNA selection in CRISPR experiments. Each crRNA was individually tested with the CH57_3927 DNA. The negative control was treated with DEPC water instead of CH57_3927 DNA, with the same other components. All experimental data are represented as mean ± standard deviation (SD) of two technical replicates.

Twelve crRNAs were designed and synthesized according to the sequence of CH57_3927 gene. The detection efficiency of each crRNA was individually tested. As shown in Fig. 2, a significant fluorescence increase was only observed in the groups using the positive plasmid as a template. Among the 12 crRNAs, crRNA2 showed the highest detection efficiency, with a slope ratio of 10.85 between the positive group and the negative control, representing superior sensitivity among the tested crRNAs (Table S1).

### Optimization of Cas12a-G4 DNAzyme visualization system

**Fig 3:**
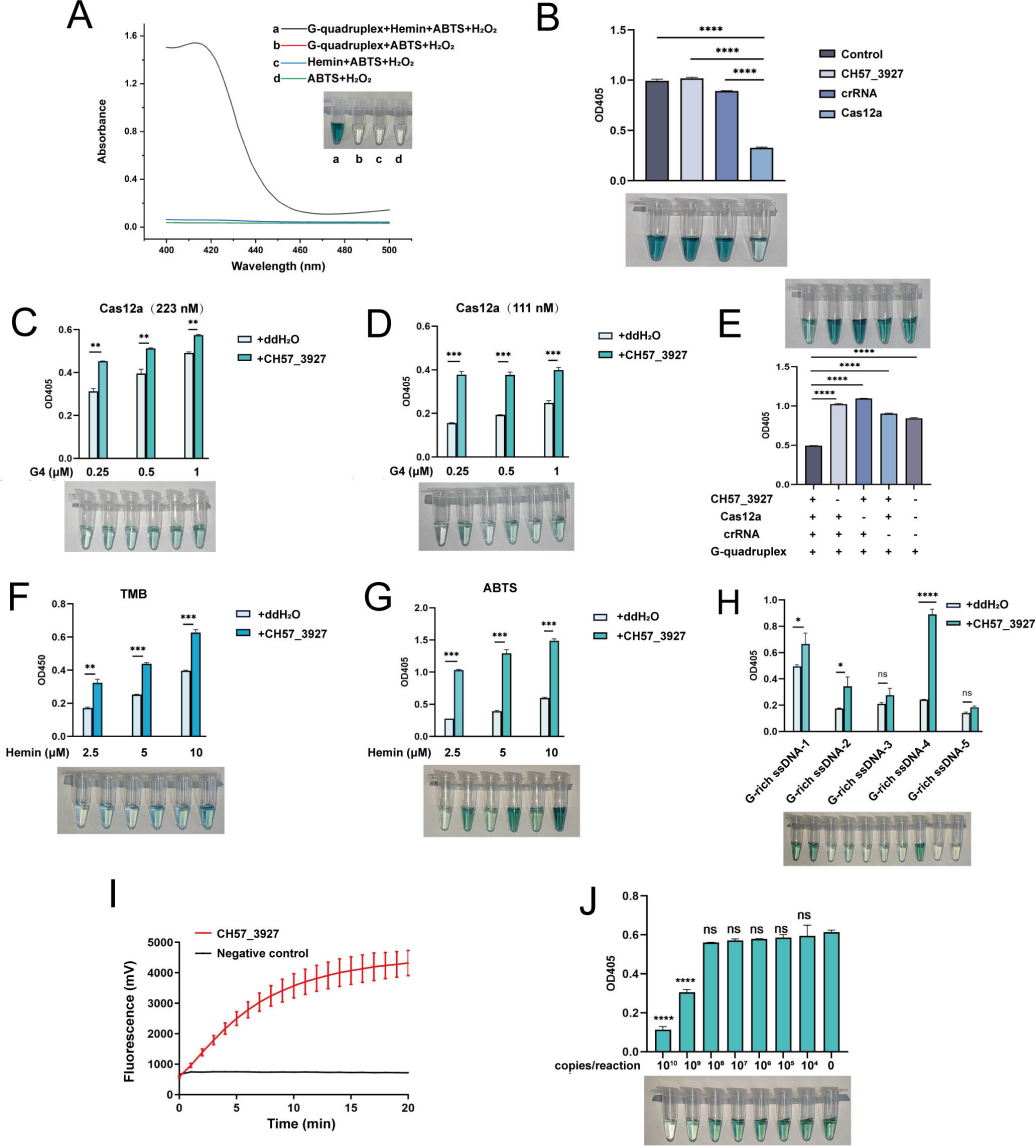
Optimization of the Cas12a-G4 visual detection system. (**A**) UV-visible absorption curve of G4 catalyzed ABTS^2−^-H_2_O_2_ color reaction. (**B**) Impact of 250 nM crRNA, 111 nM Cas12a, and 2 nM CH57_3927 (corresponding to the 20 µL CRISPR reaction system) on color development. The color reaction was conducted in a 100 µL solution containing 5 pmol G4. The control group lacked any CRISPR system components. (**C and D**) Various concentrations of Cas12a (223 nM in C and 111 nM in D) and G4 (0.25 µM, 0.5 µM, and 1 µM) on color development. (**E**) UV-visible absorbance values of G4 under various reaction conditions. (**F and G**) The influence of different concentrations of hemin (2.5 µM, 5 µM, and 10 µM) and types of color reagents (TMB in F and ABTS in G) on color development. (**H**) Impact of G4 with different sequences on color development. (**I**) Fluorescence intensity generated by the CRISPR/Cas12a system cleaving FAM-G-rich ssDNA-4-BHQ1. The negative control was treated with DEPC water instead of CH57_3927 DNA, with the same other components. Data are represented as the mean ± SD of two biological replicates. (**J**) Detection of a series of gradient dilutions of CH57_3927 using the CRISPR/Cas12a-G4 system. All experimental data in B-H are represented as mean ± standard deviation (SD) of two technical replicates. Differences among groups in B were analyzed by one-way ANOVA with Dunnett’s multiple comparisons test. Differences among groups in C, D, F, J, and H were analyzed by two-way ANOVA with Bonferroni’s multiple comparisons test. ns, not significant; *, *P* < 0.05; **, *P* < 0.01; ***, *P* < 0.001; ****, *P* < 0.0001.

The feasibility of integrating G4 DNAzyme with the Cas12a detection method was evaluated. First, the peroxidase activity of the G4/hemin DNAzyme and its ability to produce a color change for visual detection were tested. As shown in Fig. 3A, in the presence of ABTS^2–^ and H_2_O_2_ simultaneously, the peroxidase activity of G4 and hemin can catalyze the oxidation of ABTS^2–^, producing the color change from transparent to dark green. The absorption peaks ranged from 400 nm to 420 nm. It is crucial to ensure compatibility between the Cas12a detection system and the G4 DNAzyme colorimetric system. Various components in the Cas12a detection system were separately added to the G4 DNAzyme colorimetric system to evaluate their ability to inhibit color change. As shown in Fig. 3B, Cas12a significantly inhibited the color development process, which was consistent with previous studies (34, 36). This interference could be attributed to the presence of DTT in Cas12a, a reducing agent employed to protect Cas12a’s activity, which can directly neutralize ABTS-free radicals (37). It is imperative to determine the optimal Cas12a nuclease concentration that does not affect the color development process. The concentrations of the Cas12a were optimized. As shown in Fig. 3C and D, the optimal concentration of Cas12a was 111 nM and the optimal concentration of G4 was 0.25 µM, the positive and negative groups showing the most distinct color contrast. After the optimization of Cas12a, the feasibility of the integrated Cas12a-G4 detection system was further evaluated by removing a single component. As shown in Fig. 3E, G4 was degraded into fragments in the presence of CH57_3927, Cas12a, and crRNA. Consequently, the fragmented G4 failed to engage with hemin to form the G4/hemin DNA enzyme with peroxidase activity, and a very light color change, as well as a low absorption value at 405 nm, was observed.

TMB and ABTS were evaluated for their effects as substrates in visual detection. As shown in Fig. 3F and G, the color difference between positive and negative groups was more obvious using ABTS as the substrate. In addition, various concentrations of hemin in the detection system were evaluated. The positive and negative groups can be clearly distinguished by color at a hemin concentration of 2.5 µM (Fig. 3F and G).

In addition, to achieve the best detection performance, five kinds of G-rich ssDNA sequences that have been reported to be able to form G4 structures were evaluated, and G-rich ssDNA-4 performed the best as shown in Fig. 3H. We further evaluated the efficiency of Cas12a in cleaving G-rich ssDNA-4. FAM and BHQ1-labelled G-rich ssDNA-4 was used as a fluorescent reporter in the Cas12a detection system. As shown in Fig. 3I, the positive group showed a significant fluorescence increase, indicating that G-rich ssDNA-4 could be cleaved efficiently by the activated Cas12a and was suitable for use in this visual detection system.

Subsequently, the LOD of the method was evaluated using a 10-fold series dilution of the positive plasmid (ranging in concentrations from 10^10^ to 10^4^ copies/μL) as templates. The results showed that when the concentration of DNA template per reaction exceeded 10^9^ copies, an obvious color difference between the experimental and control groups was observed, indicating that the LOD of this method was 10^9^ copies/reaction (Fig. 3J).

### RAA assay establishment

**Fig 4:**
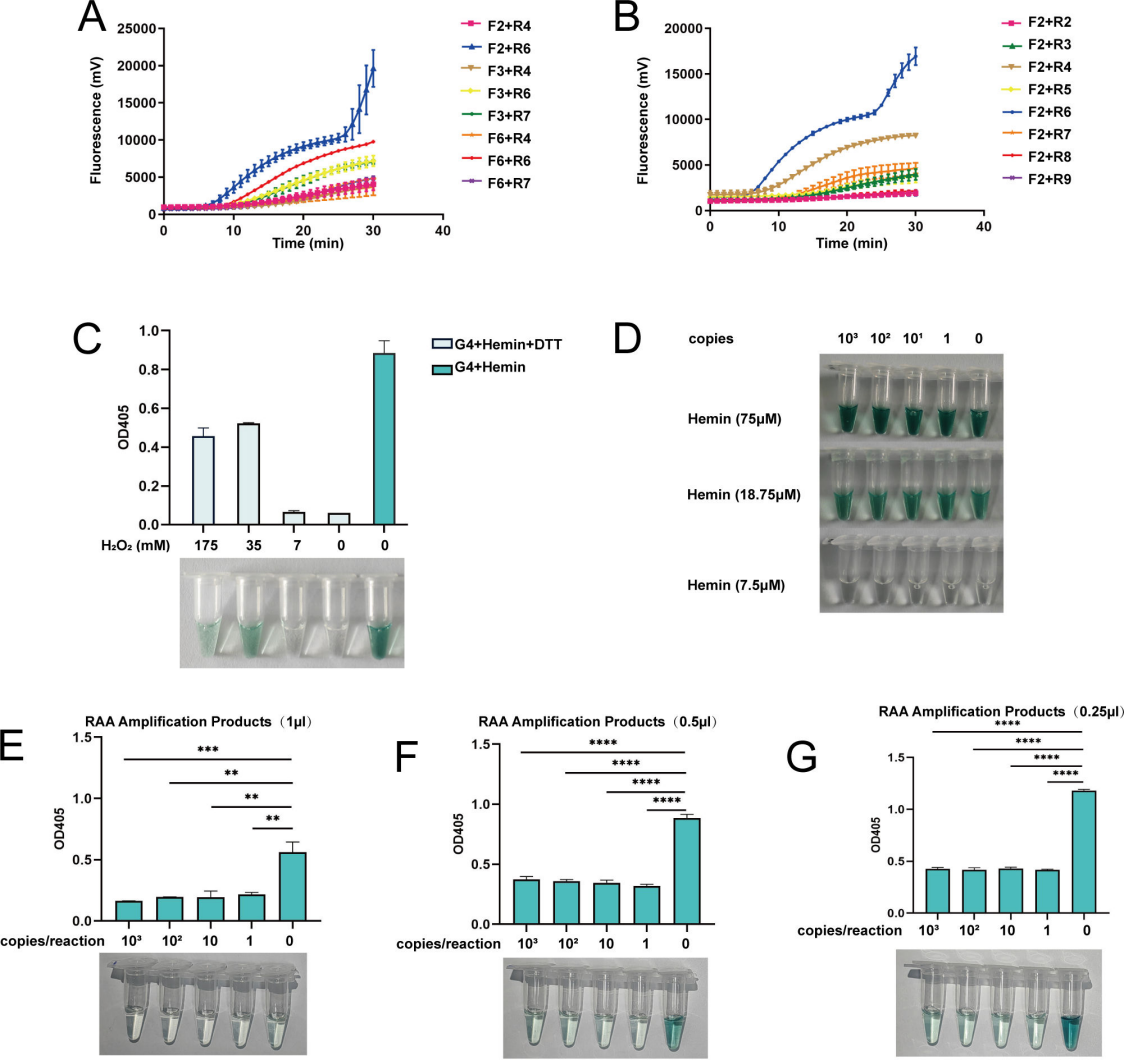
Optimization of the RAA and RCCD visual detection system. (**A and B**) Real-time fluorescence curves of 14 RAA primer combinations in RAA nucleic acid amplification experiments. (**C**) Reversal of color inhibition caused by DTT with H_2_O_2_. The colorimetric reaction was performed in a 100 µL solution comprising 50 nM G4, 500 nM hemin, and 2.5 mM DTT. (**D**) Color reaction of hemin with varied concentrations. Conduct gradient dilutions of DNA for RAA amplification and subsequently employ the amplification products for color reaction with diverse hemin concentrations. (**E through G**) Effect of different volumes of RAA amplification products (1 µL, 0.5 µL, and 0.25 µL) on color development. All experimental data in A through G are represented as mean ± standard deviation (SD) of two technical replicates. Data in E through G were analyzed by one-way ANOVA with Dunnett’s multiple comparisons test. **, *P* < 0.01; ***, *P* < 0.001; ****, *P* < 0.0001.

RAA assay was established for pre-amplification of the target sequences to improve the sensitivity of the Cas12a-G4 detection. We devised an RAA probe and a series of RAA primers both around the crRNA2 binding site. Through fluorescent RAA assays, we evaluated the amplification efficiency of various primer pairs. As a result, the primer pair F2/R6 showed the strongest fluorescence signal and the shortest positive judgment time, demonstrating its superior performance in target sequence amplification (Fig. 4A and B). The RAA amplification assay was established using this primer pair.

### Optimization of RCCD visualization system

To combine the RAA assay and the Cas12a-G4 visual detection assay, their compatibility was evaluated. The target sequences, pre-amplified by RAA, were added to the Cas12a-G4 visual detection system, and color change was observed. However, after the reaction, the color of both the positive and negative control groups did not change (Fig. S2), indicating that the reaction systems were incompatible.

We evaluated the ability of various components in the RAA system to inhibit the G4/hemin-based color development and found that DTT was the key inhibitor (Fig. 4C). We added various amounts of hydrogen peroxide (H_2_O_2_) to the DTT-inhibited system and found that the inhibitory effect could be reversed (Fig. 4C). The investigations revealed that the optimum color development enhancement was achieved when incorporating 35 mM of H_2_O_2_ (Fig. 4C).

Furthermore, we discovered that the quantity of hemin implemented also played a pivotal role in color development. High concentrations of hemin in the system could catalyze ABTS^2–^ color development independently of G4. We optimized the hemin concentration again. As shown in Fig. 4D, in the absence of G4s, 75 µM and 18.75 µM of hemin could catalyze ABTS^2–^ coloration, producing notable green products. Conversely, 7.5 µM of hemin rendered the solution almost colorless, leading us to select this concentration.

Lastly, we optimized the amount of RAA products added to the follow-up Cas12a-G4 DNAzyme colorimetric system (Fig. 4E through 4G). The results showed that introducing 0.25 µL of the RAA amplification products yielded the optimal color contrast between positive and negative samples.

### Limit of detection and specificity analysis

**Fig 5:**
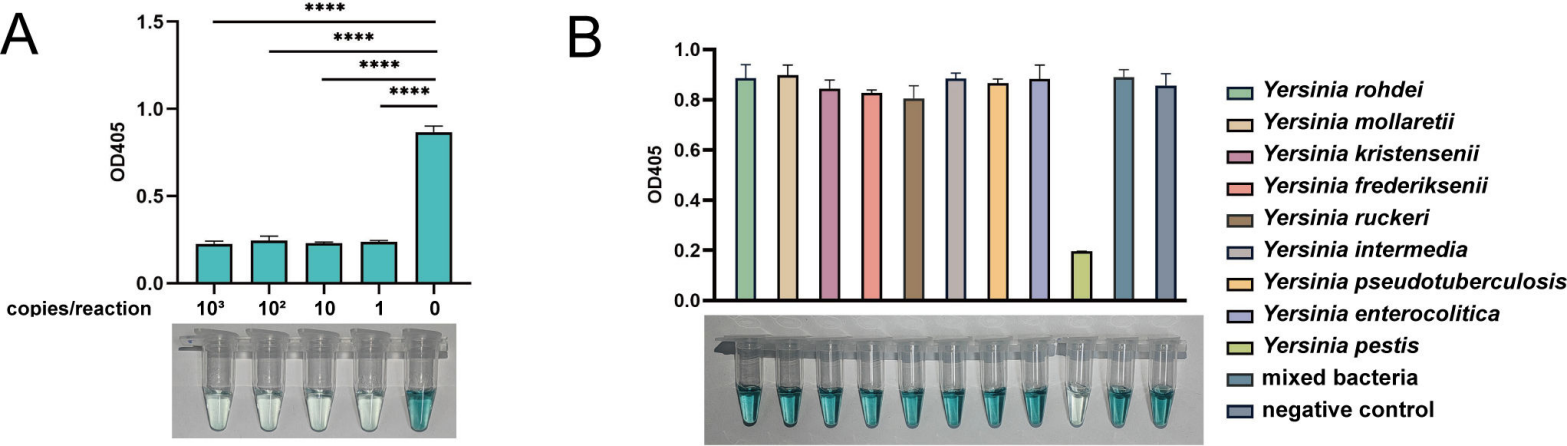
Limit of detection and specificity of *Y. pestis* detection by the RCCD visualization system. (**A**) LOD of the RCCD visualization system in detecting *Y. pestis* genomic DNA. (**B**) Specificity of the RCCD visualization system in detecting *Y. pestis* genomic DNA. Mix bacteria is a mixture of the genomes from eight bacteria, excluding *Y. pestis*. All experimental data in A and B are represented as mean ± standard deviation (SD) of two technical replicates. Differences among groups in A were analyzed using one-way ANOVA with Dunnett’s multiple comparisons test. ****, *P* < 0.0001.

To evaluate the LOD of this method for detecting *Y. pestis* DNA, we used serially diluted *Y. pestis* genomic DNA as templates, with the copy number ranging from 1 to 10^3^ copies/µL. As a result, only the negative control group showed a dark green color, indicating the LOD of the established assay as 1 copy/reaction (Fig. 5A).

The specificity of this method was also evaluated, and the established method could distinguish the genomic DNA of *Y. pestis* and the other *Yersinia* species, with only the *Y. pestis* group being colorless and the other groups being dark green (Fig. 5B). Additionally, we conducted an expanded specificity evaluation by testing six common environmental and clinical bacterial strains, including *Escherichia coli*, *Bacillus subtilis*, *Staphylococcus aureus*, *Vibrio parahaemolyticus*, *Bacillus thuringiensis,* and *Pseudomonas aeruginosa*. The results demonstrated that this method specifically detected *Y. pestis* without cross-reacting with other bacterial species (Fig. S3).

### Sensitivity and specificity evaluation using simulated clinical samples

**Fig 6:**
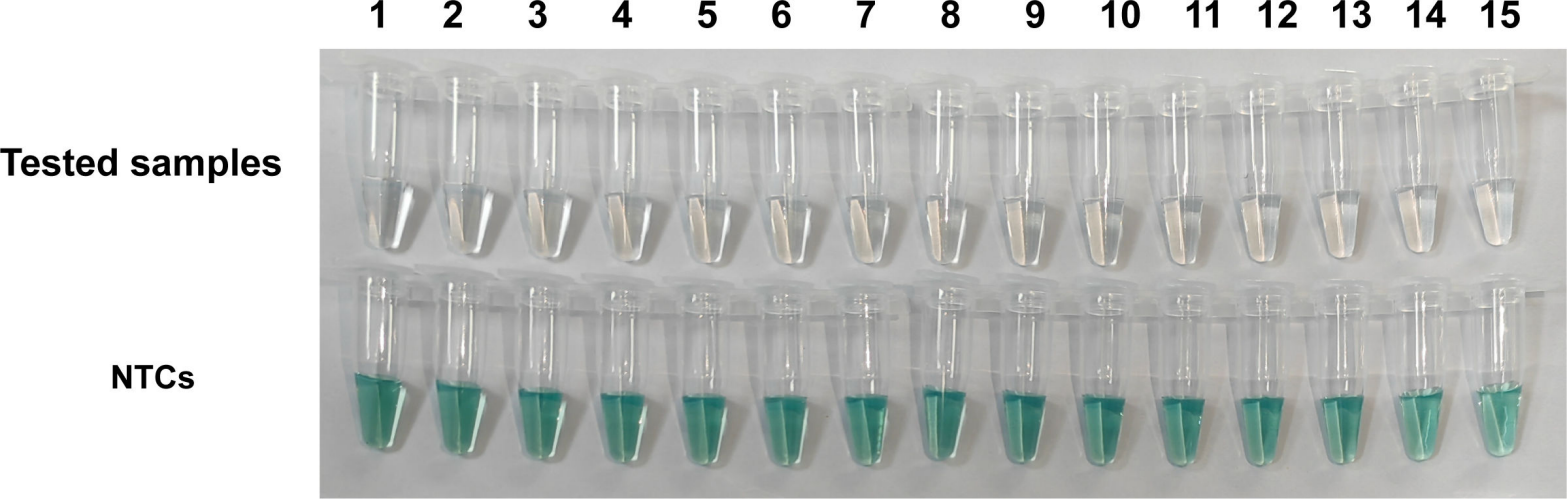
Detection of *Y. pestis* in simulated clinical samples by RCCD detection assay. Tested samples, *Y. pestis* DNA-spiked blood samples; NTCs, uninfected blood samples as no-template controls. Each test was performed in duplicate.

The sensitivity and specificity were further evaluated using 15 *Y. pestis* genomic DNA-spiked blood samples and 15 uninfected blood samples. With a concentration of 30 copies of *Y. pestis* genomic DNA per µL, all the simulated samples were detected to be positive, showing a sensitivity of 100% (15/15, Fig. 6). Meanwhile, all the uninfected blood samples were detected to be negative, showing a specificity of 100% (15/15, Fig. 6).

## DISCUSSION

*Y. pestis*, a highly pathogenic microorganism, is classified by the WHO as one of the potential bioterrorism agents. Although *Y. pestis* has been effectively controlled worldwide, cases continue to be reported in regions such as Congo, Madagascar, and Peru in recent years. These countries, with their limited economic and healthcare resources, require sensitive, rapid-response, and cost-effective detection methods to strengthen plague surveillance and prevention efforts, ensuring that large-scale epidemics do not occur again.

CRISPR detection is a novel technology, and its biggest advantage is the portability. It can be combined with various upstream amplification methods and downstream signal recognition systems. The typically used visual CRISPR detection method based on fluorescence-labeled ssDNA reporters and LF test strips greatly increases the detection cost (38). Also, it is not suitable for the detection of batch samples. In light of this, the integration of G4/hemin DNAzyme emerges as a promising and cost-effective alternative. Also, the experimental results showed good compatibility between the two systems. However, the sensitivity of CRISPR detection is low, and it is necessary to combine nucleic acid amplification technology to improve sensitivity (39). Here, we observed that a distinct signal was only discernible when the copy number of the target gene exceeded 10^9^ copies within the reaction system. A step of pre-amplification was necessary. However, our initial evaluation showed that the RAA system inhibited the G4/hemin DNAzyme reaction and was not suitable for direct use. Further analysis proved that the reducing agent DTT in the RAA system was the key inhibitor. Therefore, in the follow-up test, we added the oxidizing agent H_2_O_2_ to conquer the problem successfully. Finally, the introduction of RAA significantly improved the sensitivity, with LOD as low as 1 copy/reaction. In our study, we integrated CRISPR-Cas12a, RAA amplification, and G4 DNA enzyme to establish an advanced ELISA-like visual detection method. Compared with traditional fluorescence detection and lateral flow detection, this method has the advantages of not relying on fluorescence detection equipment, low cost, and batch detection.

The Cas12a-G4 visual detection system exhibits an inverse concentration-dependent response within the range of 10^8^ to 10^10^ template copies per reaction, wherein increasing template copies result in progressively attenuated chromogenic signals, indicating the quantitative or semi-quantitative analysis potential of the system (Fig. 3J). However, this concentration-dependent trend disappears upon integration of the RAA reaction (Fig. 4E through G), likely due to signal saturation caused by the high amplification efficiency of RAA, which impairs the quantitative capability of the system. To achieve quantitative or semi-quantitative detection, a two-stage optimization strategy is required: first, defining the linear dynamic range of the colorimetric system; second, adjusting the RAA amplification parameters (e.g., primer concentration, reaction duration) to ensure the amplified product remains within this quantifiable range. Substantial experimental optimization is necessary to implement this process.

In nucleic acid detection, the target sequence is one of the key factors that determine detection specificity. In the detection of *Y. pestis*, screening specific gene sequences for distinguishing *Y. pestis* and other pathogenic bacteria within the same genus, such as *Y. pseudotuberculosis* and *Y. enterocolitica*, is a challenge due to the high genomic sequence similarity. At least 97% of sequence homology between *Y. pestis* and *Y. pseudotuberculosis* is found in 2,976 genes (40). Specific genes like *pla, pst,* and *caf1* were used as specific identifiers for *Y. pestis* in previous studies (41–43). However, the *pla* gene has been detected in other bacteria like *Citrobacter koseri* (41) and *Escherichia coli* (42), potentially leading to false-negative results when used as a detection target. Additionally, *caf1* and *pst* are situated on plasmids pMT1 and pPCP1 (43), which are not present in all *Y. pestis* strains. Therefore, more dependable and specific molecular targets are needed to help researchers accurately identify *Y. pestis* and distinguish it from closely related species.

In our study, we compared and analyzed the homology of genome sequences of all *Yersinia* species using Mauve software. We divided the sequence of each reference genome into millions of fragments for comparative analysis between different strains. Through an in-depth comparison within *Y. pestis* strains, we screened a specific gene CH57_3927 that is consistently present in all *Y. pestis* strain genomes and only exists in *Y. pestis*. The established detection method based on this sequence did not recognize other *Yersinia* species except *Y. pestis*, proving its specificity and a reliable molecular marker, which can effectively distinguish *Y. pestis* from closely related species. As far as we know, this is the first time CH57_3927 has been used for *Y. pestis* detection, which provides a valuable reference for detecting *Y. pestis*.

One distinctive feature of this detection system is that the positive test result appears colorless, while the negative one shows a dark green color. During the experiment, failure to add the correct reagents or degradation of reagents may result in false positive outcomes. Under such circumstances, using internal quality control may be a very good solution. However, in a single colorimetric system, internal quality control cannot be compatible with the samples in a single reaction system. Therefore, we recommend incorporating positive and negative controls into the experimental system to address this issue. In addition to improving the objectivity of result interpretation, colorimetric readouts can be systematically analyzed using calibrated reference cards or smartphone-based image analysis applications. This approach effectively eliminates ambient light interference and minimizes inter-operator interpretation variances through standardized digital/analog quantification protocols.

In theory, the RCCD platform can be easily adapted for the detection of any target pathogen by simply redesigning primers and crRNAs, without altering the core mechanism. All other reaction steps and components remain unchanged, making the platform highly versatile. To validate the consistency and stability of the RCCD platform, multiple experimental replicates were performed with reagents from distinct production batches (e.g., G4, H₂O₂, DTT; Table S2), demonstrating robust reproducibility across independent tests.

There are some limitations in the present study. One limitation is the long reaction time requirement for the Cas12a-G4 colorimetric reaction. This problem likely arises from the relatively dense structure of G4, which impedes cleavage efficiency and extends the reaction time. To address this, we are exploring the use of split G-quadruplex and G-triplex structures as alternatives to G4. Another limitation of this study is the lack of clinical sample validation. Due to the unavailability of clinical samples from *Y. pestis*-infected patients, we used simulated clinical samples. However, further investigation is needed to determine whether this approach can be effectively applied to the detection of other complex sample matrices to evaluate its specificity and sensitivity. Such validation is critical for assessing the potential of this method as a reliable diagnostic tool in clinical settings.

## CONCLUSIONS

In the present study, we established an advanced ELISA-like visual detection method that integrates CRISPR-Cas12a, RAA amplification, and G4 DNAzyme for cost-effective and highly sensitive detection of *Y. pestis*. Except for the visualization detection as well as good specificity and sensitivity, the established method has a lower cost and is suitable for batch sample detection like ELISA. Moreover, it does not require complex instruments, which is convenient for rapid and on-site screening of plague outbreaks, enabling effective support for plague detection, prevention, and control at primary-level medical and health care institutions.

## SUPPLEMENTAL MATERIAL

Table S1 and S2, and Figures S1 to S3 jcm.00274-25-s0001.pdf Table S1: Reaction rates of 12 crRNAs in CRISPR experiments. Table S2: Key Reagents and Batch Information. Figure S1: Blast alignment results of CH57_3927 gene sequence. Figure S2: Effect of 5μl RAA amplification product added in 20μl Cas12a-G4 colorimetric reaction system. Figure S3: An expanded specificity assessment of RCCD visualization system in detecting *Y. pestis* Genomic DNA.

ASM does not own the copyrights to Supplemental Material that may be linked to, or accessed through, an article. The authors have granted ASM a non-exclusive, world-wide license to publish the Supplemental Material files. Please contact the corresponding author directly for reuse.

## References

[1] Rotz LD, Khan AS, Lillibridge SR, Ostroff SM, Hughes JM. 2002. Public health assessment of potential biological terrorism agents. Emerg Infect Dis 8:225–230. doi:10.3201/eid0802.010164 11897082 PMC2732458

[2] Human plague: review of regional morbidity and mortality, 2004-2009. 2009. Wkly Epidemiol Rec 6:40–45. 20151494

[3] Bertherata E. 2016. Plague around the world, 2010–2015. Wkly Epidemiol Rec 91:89–93.26922822

[4] World Health Organization. 2019. Plague around the world in 2019. Wkly Epidemiol Rec 94:289–292.

[5] World Health Organization. 2022. Plague. World Health Organization. https://www.who.int/en/news-room/fact-sheets/detail/plague.

[6] Rabaan AA, Al-Ahmed SH, Alsuliman SA, Aldrazi FA, Alfouzan WA, Haque S. 2019. The rise of pneumonic plague in Madagascar: current plague outbreak breaks usual seasonal mould. J Med Microbiol 68:292–302. doi:10.1099/jmm.0.000915 30632956

[7] World Health Organization. 2017. Madagascar plague outbreak: external situation report 14. https://www.who.int/publications/i/item/plague-outbreak-madagascar-14.

[8] Choi S, Rhie G, Jeon JH. 2020. Development of a double‐antibody sandwich ELISA for sensitive detection of Yersinia pestis . Microbiol Immunol 64:72–75. doi:10.1111/1348-0421.12751 31621104

[9] de Almeida AM, Ferreira LC. 1992. Evaluation of three serological tests for the detection of human plague in northeast Brazil. Mem Inst Oswaldo Cruz 87:87–92. doi:10.1590/s0074-02761992000100014 1308559

[10] Phillips AP, Morris BC, Hall D, Glenister M, Williams JE. 1988. Identification of encapsulated and non-encapsulated Yersinia pestis by immunofluorescence tests using polyclonal and monoclonal antibodies. Epidemiol Infect 101:59–73. doi:10.1017/s0950268800029228 3042439 PMC2249332

[11] Singh R, Pal V, Kumar M, Tripathi NK, Goel AK. 2021. Development of a PCR-lateral flow assay for rapid detection of Yersinia pestis, the causative agent of plague. Acta Trop 220:105958. doi:10.1016/j.actatropica.2021.105958 34004173

[12] Filchakova O, Dossym D, Ilyas A, Kuanysheva T, Abdizhamil A, Bukasov R. 2022. Review of COVID-19 testing and diagnostic methods. Talanta 244:123409. doi:10.1016/j.talanta.2022.123409 35390680 PMC8970625

[13] Li SY, Cheng QX, Liu JK, Nie XQ, Zhao GP, Wang J. 2024. Author correction: CRISPR-Cas12a has both cis- and trans-cleavage activities on single-stranded DNA. Cell Res 34:266–267. doi:10.1038/s41422-024-00927-2 38263279 PMC10907588

[14] Chen JS, Ma E, Harrington LB, Da Costa M, Tian X, Palefsky JM, Doudna JA. 2018. CRISPR-Cas12a target binding unleashes indiscriminate single-stranded DNase activity. Science 360:436–439. doi:10.1126/science.aar6245 29449511 PMC6628903

[15] Tian Y, Fan Z, Xu L, Cao Y, Chen S, Pan Z, Gao Y, Li H, Zheng S, Ma Y, Duan Z, Zhang X, Ren F. 2023. CRISPR/Cas13a-assisted rapid and portable HBV DNA detection for low-level viremia patients. Emerging Microbes & Infections 12:e2177088. doi:10.1080/22221751.2023.2177088 36735916 PMC9946317

[16] Xiong D, Dai W, Gong J, Li G, Liu N, Wu W, Pan J, Chen C, Jiao Y, Deng H, Ye J, Zhang X, Huang H, Li Q, Xue L, Zhang X, Tang G. 2020. Rapid detection of SARS-CoV-2 with CRISPR-Cas12a. PLoS Biol 18:e3000978. doi:10.1371/journal.pbio.3000978 33320883 PMC7737895

[17] Lin H, Liang Y, Zou L, Li B, Zhao J, Wang H, Sun J, Deng X, Tang S. 2022. Combination of isothermal recombinase-aided amplification and CRISPR-Cas12a-mediated assay for rapid detection of major severe acute respiratory syndrome coronavirus 2 variants of concern. Front Microbiol 13:945133. doi:10.3389/fmicb.2022.945133 35836420 PMC9274097

[18] Zhu D, Su T, Sun T, Qin X, Su S, Bai Y, Li F, Zhao D, Shao G, Chao J, Feng Z, Wang L. 2024. Enhancing point-of-care diagnosis of African swine fever virus (ASFV) DNA with the CRISPR-Cas12a-assisted triplex amplified assay. Anal Chem 96:5178–5187. doi:10.1021/acs.analchem.3c05364 38500378

[19] Xiao X, Lin Z, Huang X, Lu J, Zhou Y, Zheng L, Lou Y. 2021. Rapid and sensitive detection of Vibrio vulnificus using CRISPR/Cas12a combined with a recombinase-aided amplification assay. Front Microbiol 12:767315. doi:10.3389/fmicb.2021.767315 34745075 PMC8566878

[20] Hou Y, Liu X, Wang Y, Guo L, Wu L, Xia W, Zhao Y, Xing W, Chen J, Chen C. 2024. Establishment and application of a rapid visualization method for detecting Vibrio parahaemolyticus nucleic acid. Infectious Medicine 3:100111. doi:10.1016/j.imj.2024.100111 38948389 PMC11214178

[21] Yang Y, Kong X, Yang J, Xue J, Niu B, Chen Q. 2024. Rapid nucleic acid detection of listeria monocytogenes based on RAA-CRISPR Cas12a system. IJMS 25:3477. doi:10.3390/ijms25063477 38542449 PMC10971093

[22] Li Jia, Yuan T, Yang T, Xu L, Zhang L, Huang L, Cheng W, Ding S. 2018. DNA-grafted hemin with preferable catalytic properties than G-quadruplex/hemin for fluorescent miRNA biosensing. Sensors and Actuators B: Chemical 271:239–246. doi:10.1016/j.snb.2018.05.045

[23] Li J, Xiang Y, Zhang L, Huang L, Teng J, Ding S, Cheng W. 2019. Dynamic DNA self-assembly activated hemin-mimetic enzyme system for versatile fluorescent biosensing. Sensors and Actuators B: Chemical 288:757–762. doi:10.1016/j.snb.2019.03.058

[24] Ida J, Chan SK, Glökler J, Lim YY, Choong YS, Lim TS. 2019. G-quadruplexes as an alternative recognition element in disease-related target sensing. Molecules 24:1079. doi:10.3390/molecules24061079 30893817 PMC6471233

[25] Pavlov V, Xiao Y, Gill R, Dishon A, Kotler M, Willner I. 2004. Amplified chemiluminescence surface detection of DNA and telomerase activity using catalytic nucleic acid labels. Anal Chem 76:2152–2156. doi:10.1021/ac035219l 15053684

[26] Wang F, Lu CH, Liu X, Freage L, Willner I. 2014. Amplified and multiplexed detection of DNA using the dendritic rolling circle amplified synthesis of DNAzyme reporter units. Anal Chem 86:1614–1621. doi:10.1021/ac4033033 24377284

[27] Jiang HX, Liang ZZ, Ma YH, Kong DM, Hong ZY. 2016. G-quadruplex fluorescent probe-mediated real-time rolling circle amplification strategy for highly sensitive microRNA detection. Anal Chim Acta 943:114–122. doi:10.1016/j.aca.2016.09.019 27769370

[28] Yeasmin Khusbu F, Zhou X, Chen H, Ma C, Wang K. 2018. Thioflavin T as a fluorescence probe for biosensing applications. TrAC Trends in Analytical Chemistry 109:1–18. doi:10.1016/j.trac.2018.09.013

[29] Peters GM, Skala LP, Davis JT. 2016. A molecular chaperone for G4-quartet hydrogels. J Am Chem Soc 138:134–139. doi:10.1021/jacs.5b08769 26684297

[30] Yan L, Yan Y, Pei L, Wei W, Zhao J. 2014. A G-quadruplex DNA-based, label-free and ultrasensitive strategy for microRNA detection. Sci Rep 4:7400. doi:10.1038/srep07400 25492390 PMC4261168

[31] Lu J, Wang J, Hu X, Gyimah E, Yakubu S, Wang K-O, Wu X, Zhang Z-OX. 2019. Electrochemical biosensor based on tetrahedral DNA nanostructures and G-quadruplex-hemin conformation for the ultrasensitive detection of MicroRNA-21 in serum. Anal Chem 91:7353–7359. doi:10.1021/acs.analchem.9b01133 31074965

[32] Li J, Zhao J, Li S, Zhang L, Huang Y, Zhao S, Liu YM. 2016. Electrophoresis separation assisted G-quadruplex DNAzyme-based chemiluminescence signal amplification strategy on a microchip platform for highly sensitive detection of microRNA. Chem Commun 52:12806–12809. doi:10.1039/C6CC06327F PMC5079828 27711307

[33] Cai N, Li Y, Chen S, Su X. 2016. A fluorometric assay platform for caffeic acid detection based on the G-quadruplex/hemin DNAzyme. Analyst 141:4456–4462. doi:10.1039/c6an00543h 27220084

[34] Wu Z, Sun DW, Pu H. 2023. CRISPR/Cas12a and G-quadruplex DNAzyme-driven multimodal biosensor for visual detection of Aflatoxin B1. Spectrochim Acta A Mol Biomol Spectrosc 302:123121. doi:10.1016/j.saa.2023.123121 37579713

[35] Darling ACE, Mau B, Blattner FR, Perna NT. 2004. Mauve: multiple alignment of conserved genomic sequence with rearrangements. Genome Res 14:1394–1403. doi:10.1101/gr.2289704 15231754 PMC442156

[36] Chen X, Wang L, He F, Chen G, Bai L, He K, Zhang F, Xu X-O. 2021. Label-free colorimetric method for detection of Vibrio parahaemolyticus by trimming the G-quadruplex DNAzyme with CRISPR/Cas12a . Anal Chem 93:14300–14306. doi:10.1021/acs.analchem.1c03468 34645259

[37] Baňasová M, Valachová K, Juránek I, Šoltés L. 2014. Dithiols as more effective than monothiols in protecting biomacromolecules from free-radical-mediated damage: in vitro oxidative degradation of high-molar-mass hyaluronan. Chem Zvesti 68:1428–1434. doi:10.2478/s11696-014-0591-1

[38] Yu H, Jing W, Cheng X. 2023. CRISPR-Cas- and aptamer-based systems for diagnosing pathogens: a review. Zoonoses 3:22. doi:10.15212/ZOONOSES-2023-0008

[39] Li X, Zhu S, Zhang X, Ren Y, He J, Zhou J, Yin L, Wang G, Zhong T, Wang L, Xiao Y, Zhu C, Yin C, Yu X. 2023. Advances in the application of recombinase-aided amplification combined with CRISPR-Cas technology in quick detection of pathogenic microbes. Front Bioeng Biotechnol 11:1215466. doi:10.3389/fbioe.2023.1215466 37720320 PMC10502170

[40] Chain PSG, Carniel E, Larimer FW, Lamerdin J, Stoutland PO, Regala WM, Georgescu AM, Vergez LM, Land ML, Motin VL, Brubaker RR, Fowler J, Hinnebusch J, Marceau M, Medigue C, Simonet M, Chenal-Francisque V, Souza B, Dacheux D, Elliott JM, Derbise A, Hauser LJ, Garcia E. 2004. Insights into the evolution of Yersinia pestis through whole-genome comparison with Yersinia pseudotuberculosis. Proc Natl Acad Sci USA 101:13826–13831. doi:10.1073/pnas.0404012101 15358858 PMC518763

[41] Armougom F, Bitam I, Croce O, Merhej V, Barassi L, Nguyen T-T, La Scola B, Raoult D. 2016. Genomic insights into a new citrobacter koseri strain revealed gene exchanges with the virulence-associated Yersinia pestis pPCP1 plasmid. Front Microbiol 7:340. doi:10.3389/fmicb.2016.00340 27014253 PMC4793686

[42] Hänsch S, Cilli E, Catalano G, Gruppioni G, Bianucci R, Stenseth NC, Bramanti B, Pallen MJ. 2015. The pla gene, encoding plasminogen activator, is not specific to Yersinia pestis. BMC Res Notes 8:535. doi:10.1186/s13104-015-1525-x 26438258 PMC4593223

[43] Bai Y, Motin V, Enscore RE, Osikowicz L, Rosales Rizzo M, Hojgaard A, Kosoy M, Eisen RJ. 2020. Pentaplex real-time PCR for differential detection of Yersinia pestis and Y. pseudotuberculosis and application for testing fleas collected during plague epizootics. Microbiologyopen 9:e1105. doi:10.1002/mbo3.1105 32783386 PMC7568250

