# Supplementary material for: An ELISA-like sensitive and visual detection system targeting Yersinia pestis based on CRISPR/Cas12a and DNAzyme
Source: J Clin Microbiol. 2025 Jul 24;63(8):e00274-25. doi: 10.1128/jcm.00274-25 (PMC12345247; doi:10.1128/jcm.00274-25)
Supplement: Table S1 and S2, and Figures S1 to S3 — Table S1: Reaction rates of 12 crRNAs in CRISPR experiments. Table S2: Key Reagents and Batch Information. Figure S1: Blast alignment results of CH57_3927 gene sequence. Figure S2: Effect of 5μl RAA amplification product added in 20μl Cas12a-G4 colorimetric reaction system. Figure S3: An expanded specificity assessment of RCCD visualization system in detecting Y. pestis Genomic DNA. [file jcm.00274-25-s0001.pdf]

Supporting information

An ELISA-like sensitive and visual detection system targeting *Yersinia pestis* based on CRISPR/Cas12a and DNAzyme

Yingqing Mao[1†], Ruichen Lv [1†], Hao Shao[2†], Yong Zhao[3], Junhu Wang[1], Qiong Chen[1], Haiming Yi[1], Yixin Ge[1], Hongming Wang[1], Yuexi Li[1*], Yong Qi[1*]

1 Huadong Research Institute for Medicine and Biotechniques, Nanjing 210002, China
2 Teachers College, Columbia University, Manhattan, New York 10027, United States
3 State Key Laboratory of Pathogen and Biosecurity, Beijing Institute of Microbiology and Epidemiology, Beijing 100071, China

Table S1. Reaction rates of 12 crRNAs in CRISPR experiments. The reaction rate is determined by the ratio of the slopes of two linear fitting curves.

	crRNA1	crRNA2	crRNA3	crRNA4	crRNA5	crRNA6	crRNA7	crRNA8	crRNA9	crRNA10	crRNA11	crRNA12
Slope(CH57_3927)	31.54	86.82	100.00	75.54	48.85	94.17	33.59	44.90	25.08	28.09	29.00	32.65
Slope(Negative control)	14.97	8.21	15.36	12.59	19.69	21.22	11.52	14.77	14.36	18.03	16.48	13.35
Slope(CH57_3927)/Slope(Negative control)	2.11	10.58	6.51	6.00	2.48	4.44	2.92	3.04	1.75	1.56	1.76	2.45

Table S2. Key Reagents and Batch Information.

Reagents	Lot Numbers
RAA nucleic acid amplification kit	BG2JE0，BG72B0 and BF8ED0
LbCas12a Nuclease	C2407001，C2403002 and C2308007
H_2O_2	20230227 and 20230311
EL-ABTS Chromogenic Reagent kit	K724DA0012 and K115DA0002
Hemin	2230804001 and 24230804004

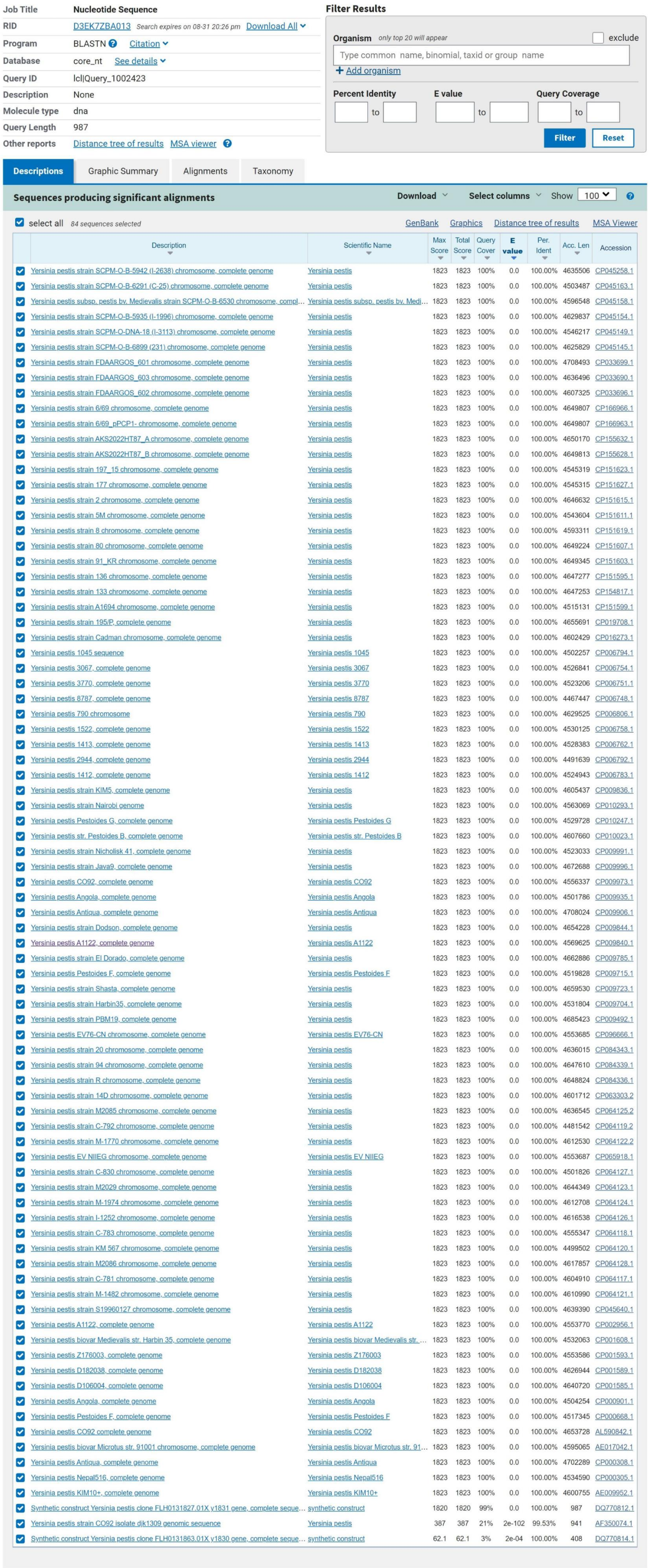

Job Title	Nucleotide Sequence
RID	D3EK7ZBA013 Search expires on 08-31 20:26 pm Download All
Program	BLASTN Citation
Database	core_nt See details
Query ID	lcl|Query_1002423
Description	None
Molecule type	dna
Query Length	987
Other reports	Distance tree of results MSA viewer

Description	Scientific Name	Max Score	Total Score	Query Cover	E value	Per. Ident	Acc. Len	Accession
Yersinia pestis strain SCPM-O-B-5942 (I-2638) chromosome, complete genome	Yersinia pestis	1823	1823	100%	0.0	100.00%	4635506	CP045258.1
Yersinia pestis strain SCPM-O-B-6291 (C-25) chromosome, complete genome	Yersinia pestis	1823	1823	100%	0.0	100.00%	4503487	CP045163.1
Yersinia pestis subsp. pestis bv. Medievalis strain SCPM-O-B-6530 chromosome, compl…	Yersinia pestis subsp. pestis bv. Medi…	1823	1823	100%	0.0	100.00%	4596548	CP045158.1
Yersinia pestis strain SCPM-O-B-5935 (I-1996) chromosome, complete genome	Yersinia pestis	1823	1823	100%	0.0	100.00%	4629837	CP045154.1
Yersinia pestis strain SCPM-O-DNA-18 (I-3113) chromosome, complete genome	Yersinia pestis	1823	1823	100%	0.0	100.00%	4546217	CP045149.1
Yersinia pestis strain SCPM-O-B-6899 (231) chromosome, complete genome	Yersinia pestis	1823	1823	100%	0.0	100.00%	4625829	CP045145.1
Yersinia pestis strain FDAARGOS_601 chromosome, complete genome	Yersinia pestis	1823	1823	100%	0.0	100.00%	4708493	CP033699.1
Yersinia pestis strain FDAARGOS_603 chromosome, complete genome	Yersinia pestis	1823	1823	100%	0.0	100.00%	4636496	CP033690.1
Yersinia pestis strain FDAARGOS_602 chromosome, complete genome	Yersinia pestis	1823	1823	100%	0.0	100.00%	4607325	CP033696.1
Yersinia pestis strain 6/69 chromosome, complete genome	Yersinia pestis	1823	1823	100%	0.0	100.00%	4649807	CP166966.1
Yersinia pestis strain 6/69_pPCP1- chromosome, complete genome	Yersinia pestis	1823	1823	100%	0.0	100.00%	4649807	CP166963.1
Yersinia pestis strain AKS2022HT87_A chromosome, complete genome	Yersinia pestis	1823	1823	100%	0.0	100.00%	4650170	CP155632.1
Yersinia pestis strain AKS2022HT87_B chromosome, complete genome	Yersinia pestis	1823	1823	100%	0.0	100.00%	4649813	CP155628.1
Yersinia pestis strain 197_15 chromosome, complete genome	Yersinia pestis	1823	1823	100%	0.0	100.00%	4545319	CP151623.1
Yersinia pestis strain 177 chromosome, complete genome	Yersinia pestis	1823	1823	100%	0.0	100.00%	4545315	CP151627.1
Yersinia pestis strain 2 chromosome, complete genome	Yersinia pestis	1823	1823	100%	0.0	100.00%	4646632	CP151615.1
Yersinia pestis strain 5M chromosome, complete genome	Yersinia pestis	1823	1823	100%	0.0	100.00%	4543604	CP151611.1
Yersinia pestis strain 8 chromosome, complete genome	Yersinia pestis	1823	1823	100%	0.0	100.00%	4593311	CP151619.1
Yersinia pestis strain 80 chromosome, complete genome	Yersinia pestis	1823	1823	100%	0.0	100.00%	4649224	CP151607.1
Yersinia pestis strain 91_KR chromosome, complete genome	Yersinia pestis	1823	1823	100%	0.0	100.00%	4649345	CP151603.1
Yersinia pestis strain 136 chromosome, complete genome	Yersinia pestis	1823	1823	100%	0.0	100.00%	4647277	CP151595.1
Yersinia pestis strain 133 chromosome, complete genome	Yersinia pestis	1823	1823	100%	0.0	100.00%	4647253	CP154817.1
Yersinia pestis strain A1694 chromosome, complete genome	Yersinia pestis	1823	1823	100%	0.0	100.00%	4515131	CP151599.1
Yersinia pestis strain 195/P, complete genome	Yersinia pestis	1823	1823	100%	0.0	100.00%	4655691	CP019708.1
Yersinia pestis strain Cadman chromosome, complete genome	Yersinia pestis	1823	1823	100%	0.0	100.00%	4602429	CP016273.1
Yersinia pestis 1045 sequence	Yersinia pestis 1045	1823	1823	100%	0.0	100.00%	4502257	CP006794.1
Yersinia pestis 3067, complete genome	Yersinia pestis 3067	1823	1823	100%	0.0	100.00%	4526841	CP006754.1
Yersinia pestis 3770, complete genome	Yersinia pestis 3770	1823	1823	100%	0.0	100.00%	4523206	CP006751.1
Yersinia pestis 8787, complete genome	Yersinia pestis 8787	1823	1823	100%	0.0	100.00%	4467447	CP006748.1
Yersinia pestis 790 chromosome	Yersinia pestis 790	1823	1823	100%	0.0	100.00%	4629525	CP006806.1
Yersinia pestis 1522, complete genome	Yersinia pestis 1522	1823	1823	100%	0.0	100.00%	4530125	CP006758.1
Yersinia pestis 1413, complete genome	Yersinia pestis 1413	1823	1823	100%	0.0	100.00%	4528383	CP006762.1
Yersinia pestis 2944, complete genome	Yersinia pestis 2944	1823	1823	100%	0.0	100.00%	4491639	CP006792.1
Yersinia pestis 1412, complete genome	Yersinia pestis 1412	1823	1823	100%	0.0	100.00%	4524943	CP006783.1
Yersinia pestis strain KIM5, complete genome	Yersinia pestis	1823	1823	100%	0.0	100.00%	4605437	CP009836.1
Yersinia pestis strain Nairobi genome	Yersinia pestis	1823	1823	100%	0.0	100.00%	4563069	CP010293.1
Yersinia pestis Pestoides G, complete genome	Yersinia pestis Pestoides G	1823	1823	100%	0.0	100.00%	4529728	CP010247.1
Yersinia pestis str. Pestoides B, complete genome	Yersinia pestis str. Pestoides B	1823	1823	100%	0.0	100.00%	4607660	CP010023.1
Yersinia pestis strain Nicholisk 41, complete genome	Yersinia pestis	1823	1823	100%	0.0	100.00%	4523033	CP009991.1
Yersinia pestis strain Java9, complete genome	Yersinia pestis	1823	1823	100%	0.0	100.00%	4672688	CP009996.1
Yersinia pestis CO92, complete genome	Yersinia pestis CO92	1823	1823	100%	0.0	100.00%	4556337	CP009973.1
Yersinia pestis Angola, complete genome	Yersinia pestis Angola	1823	1823	100%	0.0	100.00%	4501786	CP009935.1
Yersinia pestis Antiqua, complete genome	Yersinia pestis Antiqua	1823	1823	100%	0.0	100.00%	4708024	CP009906.1
Yersinia pestis strain Dodson, complete genome	Yersinia pestis	1823	1823	100%	0.0	100.00%	4654228	CP009844.1
Yersinia pestis A1122, complete genome	Yersinia pestis A1122	1823	1823	100%	0.0	100.00%	4569625	CP009840.1
Yersinia pestis strain El Dorado, complete genome	Yersinia pestis	1823	1823	100%	0.0	100.00%	4662886	CP009785.1
Yersinia pestis Pestoides F, complete genome	Yersinia pestis Pestoides F	1823	1823	100%	0.0	100.00%	4519828	CP009715.1
Yersinia pestis strain Shasta, complete genome	Yersinia pestis	1823	1823	100%	0.0	100.00%	4659530	CP009723.1
Yersinia pestis strain Harbin35, complete genome	Yersinia pestis	1823	1823	100%	0.0	100.00%	4531804	CP009704.1
Yersinia pestis strain PBM19, complete genome	Yersinia pestis	1823	1823	100%	0.0	100.00%	4685423	CP009492.1
Yersinia pestis EV76-CN chromosome, complete genome	Yersinia pestis EV76-CN	1823	1823	100%	0.0	100.00%	4553685	CP096666.1
Yersinia pestis strain 20 chromosome, complete genome	Yersinia pestis	1823	1823	100%	0.0	100.00%	4636015	CP084343.1
Yersinia pestis strain 94 chromosome, complete genome	Yersinia pestis	1823	1823	100%	0.0	100.00%	4647610	CP084339.1
Yersinia pestis strain R chromosome, complete genome	Yersinia pestis	1823	1823	100%	0.0	100.00%	4648824	CP084336.1
Yersinia pestis strain 14D chromosome, complete genome	Yersinia pestis	1823	1823	100%	0.0	100.00%	4601712	CP063303.2
Yersinia pestis strain M2085 chromosome, complete genome	Yersinia pestis	1823	1823	100%	0.0	100.00%	4636545	CP064125.2
Yersinia pestis strain C-792 chromosome, complete genome	Yersinia pestis	1823	1823	100%	0.0	100.00%	4481542	CP064119.2
Yersinia pestis strain M-1770 chromosome, complete genome	Yersinia pestis	1823	1823	100%	0.0	100.00%	4612530	CP064122.2
Yersinia pestis EV NIIEG chromosome, complete genome	Yersinia pestis EV NIIEG	1823	1823	100%	0.0	100.00%	4553687	CP065918.1
Yersinia pestis strain C-830 chromosome, complete genome	Yersinia pestis	1823	1823	100%	0.0	100.00%	4501826	CP064127.1
Yersinia pestis strain M2029 chromosome, complete genome	Yersinia pestis	1823	1823	100%	0.0	100.00%	4644349	CP064123.1
Yersinia pestis strain M-1974 chromosome, complete genome	Yersinia pestis	1823	1823	100%	0.0	100.00%	4612708	CP064124.1
Yersinia pestis strain I-1252 chromosome, complete genome	Yersinia pestis	1823	1823	100%	0.0	100.00%	4616538	CP064126.1
Yersinia pestis strain C-783 chromosome, complete genome	Yersinia pestis	1823	1823	100%	0.0	100.00%	4555347	CP064118.1
Yersinia pestis strain KM 567 chromosome, complete genome	Yersinia pestis	1823	1823	100%	0.0	100.00%	4499502	CP064120.1
Yersinia pestis strain M2086 chromosome, complete genome	Yersinia pestis	1823	1823	100%	0.0	100.00%	4617857	CP064128.1
Yersinia pestis strain C-781 chromosome, complete genome	Yersinia pestis	1823	1823	100%	0.0	100.00%	4604910	CP064117.1
Yersinia pestis strain M-1482 chromosome, complete genome	Yersinia pestis	1823	1823	100%	0.0	100.00%	4610990	CP064121.1
Yersinia pestis strain S19960127 chromosome, complete genome	Yersinia pestis	1823	1823	100%	0.0	100.00%	4639390	CP045640.1
Yersinia pestis A1122, complete genome	Yersinia pestis A1122	1823	1823	100%	0.0	100.00%	4553770	CP002956.1
Yersinia pestis biovar Medievalis str. Harbin 35, complete genome	Yersinia pestis biovar Medievalis str. …	1823	1823	100%	0.0	100.00%	4532063	CP001608.1
Yersinia pestis Z176003, complete genome	Yersinia pestis Z176003	1823	1823	100%	0.0	100.00%	4553586	CP001593.1
Yersinia pestis D182038, complete genome	Yersinia pestis D182038	1823	1823	100%	0.0	100.00%	4626944	CP001589.1
Yersinia pestis D106004, complete genome	Yersinia pestis D106004	1823	1823	100%	0.0	100.00%	4640720	CP001585.1
Yersinia pestis Angola, complete genome	Yersinia pestis Angola	1823	1823	100%	0.0	100.00%	4504254	CP000901.1
Yersinia pestis Pestoides F, complete genome	Yersinia pestis Pestoides F	1823	1823	100%	0.0	100.00%	4517345	CP000668.1
Yersinia pestis CO92 complete genome	Yersinia pestis CO92	1823	1823	100%	0.0	100.00%	4653728	AL590842.1
Yersinia pestis biovar Microtus str. 91001 chromosome, complete genome	Yersinia pestis biovar Microtus str. 91…	1823	1823	100%	0.0	100.00%	4595065	AE017042.1
Yersinia pestis Antiqua, complete genome	Yersinia pestis Antiqua	1823	1823	100%	0.0	100.00%	4702289	CP000308.1
Yersinia pestis Nepal516, complete genome	Yersinia pestis Nepal516	1823	1823	100%	0.0	100.00%	4534590	CP000305.1
Yersinia pestis KIM10+, complete genome	Yersinia pestis KIM10+	1823	1823	100%	0.0	100.00%	4600755	AE009952.1
Synthetic construct Yersinia pestis clone FLH0131827.01X y1831 gene, complete seque…	synthetic construct	1820	1820	99%	0.0	100.00%	987	DQ770812.1
Yersinia pestis strain CO92 isolate djk1309 genomic sequence	Yersinia pestis	387	387	21%	2e-102	99.53%	941	AF350074.1
Synthetic construct Yersinia pestis clone FLH0131863.01X y1830 gene, complete seque…	synthetic construct	62.1	62.1	3%	2e-04	100.00%	408	DQ770814.1

Figure S1. Blast alignment results of CH57_3927 gene sequence.

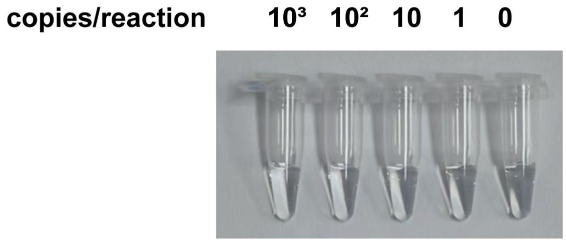

Figure S2. Effect of 5µl RAA amplification product added in 20µl Cas12a-G4 colorimetric reaction system.

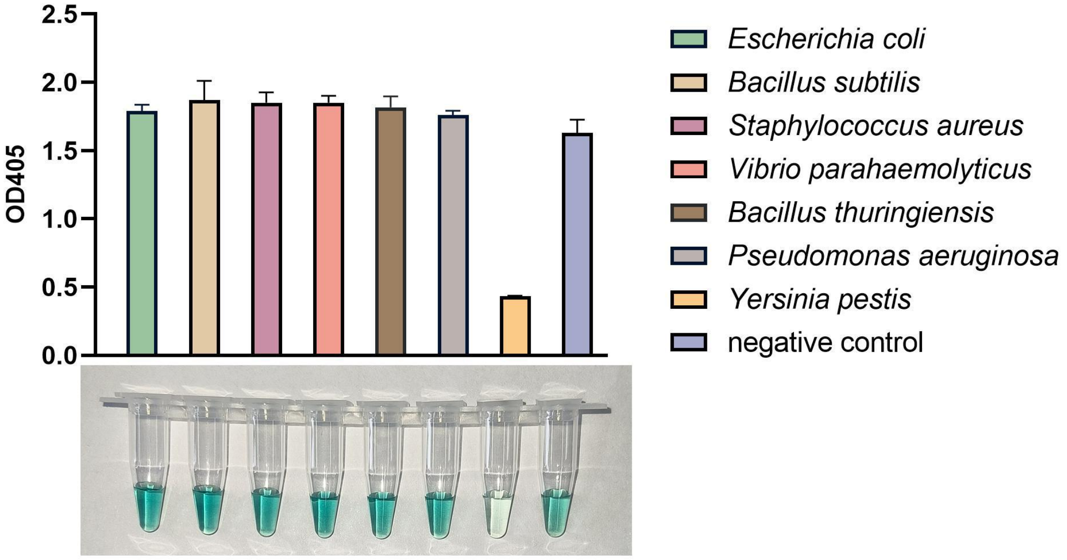

Figure S3. An expanded specificity assessment of RCCD visualization system in detecting *Y. pestis* Genomic DNA. All experimental data are represented as mean ± standard deviation (SD) of two technical replicates. .
